# Identification of Known and Novel MicroRNAs in Raspberry Organs Through High-Throughput Sequencing

**DOI:** 10.3389/fpls.2020.00728

**Published:** 2020-06-03

**Authors:** Gengxuan Yan, Jie Zhang, Meng Jiang, Xince Gao, Hongyi Yang, Lili Li

**Affiliations:** ^1^Key Laboratory of Saline-alkali Vegetation Ecology Restoration (Northeast Forestry University), Ministry of Education, Harbin, China; ^2^College of Life Science, Northeast Forestry University, Harbin, China; ^3^Institute of Forestry Science of Heilongjiang Province, Harbin, China

**Keywords:** small RNA, raspberry, high-throughput sequencing, miRNA target, molecular function

## Abstract

MicroRNAs (miRNAs) are a class of small endogenous RNAs that play important regulatory roles in plants by negatively affecting gene expression. Studies on the identification of miRNAs and their functions in various plant species and organs have significantly contributed to plant development research. In the current study, we utilized high-throughput sequencing to detect the miRNAs in the root, stem, and leaf tissues of raspberry (*Rubus idaeus*). A total of more than 35 million small RNA reads ranging in size from 18 to 35 nucleotides were obtained, with 147 known miRNAs and 542 novel miRNAs identified among the three organs. Sequence verification and the relative expression profiles of the six known miRNAs were investigated by stem-loop quantitative real-time PCR. Furthermore, the potential target genes of the known and novel miRNAs were predicted and subjected to Gene Ontology (GO) and Kyoto Encyclopedia of Genes and Genomes pathway annotation. Enrichment analysis of the GO-associated biological processes and molecular functions revealed that these target genes were potentially involved in a wide range of metabolic pathways and developmental processes. Moreover, the miRNA target prediction revealed that most of the targets predicted as transcription factor-coding genes are involved in cellular and metabolic processes. This report is the first to identify miRNAs in raspberry. The detected miRNAs were analyzed by cluster analysis according to their expression, which revealed that these conservative miRNAs are necessary for plant functioning. The results add novel miRNAs to the raspberry transcriptome, providing a useful resource for the further elucidation of the functional roles of miRNAs in raspberry growth and development.

## Introduction

Raspberry fruit has a high nutritive value, excellent flavor, and multiple health components, including ellagic acid, bioflavonoids, superoxide dismutase, and salicylic acid (Bobinaite et al., 2012). Raspberries are also used in cosmetics, medicine, health care, and other fields (Oomah et al., 2000; Bowen-Forbes et al., 2010). Over the past decade, consumer demand for raspberries has increased considerably, and the area of raspberry (*Rubus idaeus*) planting area has extended rapidly. There are two fruiting types in raspberries: floricane and primocane-fruiting (Dale, 2008). In primocane fruiting cultivars, the flowers bloom during late summer and early autumn, and fruiting can occur in the first year of development (Carew et al., 2015). In floricane fruiting cultivars, raspberries are mostly biennial canes (stems) on a long-lived perennial rootstock, which initially forms from seedling establishment or the production of root suckers. Sterile first-year stems (primocanes) develop from buds at or below the ground surface and generally produce only leaves (Whitney, 1982). During the second year, lateral branches, known as floricanes, develop from the axils of the primocanes, and produce both leaves and fruits.

A “typical” raspberry rootstock system consist at least one floricane and several primocanes (Anderson, 1980). Though the raspberry plant is perennial, its canes (stems) are biennial. Each cane passes through a well-defined sequence of seasonal phases during its 2-year lifespan. In this way, the raspberry is an ideal plant for studying the effects of environmental factors on the processes of growth, flower initiation, and the induction and breaking of dormancy of vegetative and flowering apices (Hudson, 1959). Most raspberry cultivars take 3 years from planting to reach their full productive potential. Currently, seedling development of raspberries, especially in nutrient-poor or salt-stress soil is still a problem. An improved understanding of the molecular mechanisms involved in the growth of raspberry would be of great significance for the development of raspberry cultivation technology and could help increase the global value and supply of this popular product.

Small silencing RNAs are endogenous and exogenous RNAs that play important gene-regulatory roles in eukaryotes (Bartel, 2004). A previous study demonstrated that microRNAs (miRNAs, 19–35 nt in length) and short interfering RNAs (siRNAs, mostly 22–25 nt in length), which are significant in plants and animals, are the two main classes of small silencing RNAs (Gebert and Macrae, 2019; Sun et al., 2019). The miRNAs mediate translational repression or degradation as components of the regulatory pathways involved in plant growth and development (He and Hannon, 2004; Valencia-Sanchez et al., 2006; Shanker and Maheswari, 2017).

In recent years, miRNAs have been identified in some plants species through cloning and computational approaches (Saliminejad et al., 2019; Smoczynska et al., 2019), and it has also been shown that miRNAs may be predicted using modern machine learning approaches (Parveen et al., 2019; Esposito et al., 2020). Recent reports have identified hundreds of miRNAs in several species, including *Fragaria vesca* (Han et al., 2019), cardamom (Anjali et al., 2019), sweet cherry (Wang et al., 2019), and Brazilian pine (Galdino et al., 2019) through high-throughput sequencing. Currently, 38,589 entries representing hairpin precursor miRNAs and 48,860 mature miRNAs have been discovered from 271 organisms, and these are available in the public miRNA database miRbase (Release 22.1, October 2018)^1^ (Kozomara et al., 2018). High-throughput sequencing has been widely used to identify conserved and novel miRNAs in plants, which has enlarged the realm of miRNA research (Simsek et al., 2017; Jiu et al., 2019). Studies that predict miRNA target genes provide an alternative method for identifying the regulatory functions of miRNAs through downstream processes. Related analysis between miRNAs and their targets is an efficient method of evaluating the target genes based on high homology (Wang et al., 2012). Nevertheless, there are few reports on miRNAs in raspberry, and thus, the functions or molecular mechanisms of these miRNAs are equivocal.miRNAs are important regulatory factors in plants. miRNAs not only participate in guiding seed germination, seedling differentiation, plant growth, development, and the morphological processes of the tissues and organs but also regulate hormone signal transmission and resistance to adverse environments (Song et al., 2019). The miRNA is cut and edited by a dicer-like protein (DCL) process. DICER-LIKE enzymes (DCLs) act during miRNA metabolism (DCL1), viral resistance (DCL2), transcriptional silencing (DCL3), post-transcriptional silencing and tasiRNA metabolism (DCL4). DCL1 is the key enzyme of the miRNA biosynthesis pathway and is involved in the transcription of small-interfering RNAs, playing an important role in the defense against DNA viruses and bacteria (Liu et al., 2009). DCL1 also suppresses the silencing of antiviral RNA by negatively regulating the expression of DCL4 and DCL3 (Liu et al., 2009; Fukudome and Fukuhara, 2017; Singh et al., 2019). The products are then incorporated into functional complexes that are known as “RISC,” which stands for RNA-induced silencing complexes that exist temporarily (Chendrimada et al., 2007; Zheng, 2017). Usually, one strand of the miRNA/miRNA^∗^ duplex in a RISC will be retained, whereas the other strand degrades (Kobayashi and Tomari, 2016). To understand the role of miRNA and miRNA^∗^-strands in plant growth, we need to elucidate the developmental processes of raspberry from a new perspective (Singh et al., 2018).

In this study, we used high-throughput sequencing to identify known miRNAs, predicted the novel small RNAs, and conducted count-based expression profiling of miRNAs. We identified miRNAs that were highly expressed in different raspberry organs and excavated the miRNA targets and compared their relative expression among all of the samples by quantitative real-time (qRT)-PCR analysis. Furthermore, Gene Ontology (GO) enrichment and pathway enrichment of miRNA target genes were conducted. Overall, several known and non-annotated miRNAs showed remarkable differences in the organs, and their tissue-specific functions were analyzed to evaluate their influence on seedling growth. Significantly differentially expressed miRNA in the roots and leaves may control the growth of plant leaves or plant photosynthesis, while the significantly differentially expressed miRNA in the roots and stems may control plant elongation. This study describes an inventory of miRNAs, explores the putative functions and provides a foundation for future studies in raspberry growth.

## Materials and Methods

### Plant Material and Sample Preparation

*Rubus idaeus* seeds (cv. Heritage) kept at the Key Laboratory of Saline-alkali Vegetation Ecology Restoration were used in this study. Seeds were disinfected with 10% NaClO for 20 min and rinsed several times with sterile water. After soaking in 100 mg/L gibberellic acid (GA_3_) solution for 4 h, the seeds were sown in a soil matrix (90% turfy soil, 5% vermiculite and 5% fermented pine needles) and grown in a controlled growth room (25°C, 16/8 h, day/night, 1600 lux). Roots, stems, and leaves were collected during the second squaring stage of 1-year-old raspberry plants and stored at −80°C. Three individual biological repeats for each tissue were prepared.

### Construction of Small RNA Libraries

More than 100 ng of total RNA, extracted using TRIzol (Rio et al., 2010) from the root (MR), stem (MS), and leaf (ML) tissues, was required for library construction. The quantity and purity of the total RNA were assessed using the Agilent 2100 Bioanalyzer system (Santa Clara, CA, United States) and denaturing gel electrophoresis. RNA segments of different sizes were separated through PAGE gels to recover between 18- and 35-nucleotide stripes. Then, the 3′ adapters were added by T4 RNA ligase and the 36–44 nt RNAs were enriched, then the 5′ adapters were ligated to the RNAs. Next, these products were reverse transcribed and amplified by PCR. The 140–160-bp-sized PCR products containing adapters were enriched to generate a cDNA library. We then performed single-end sequencing on an Illumina HiSeq2500 at the Genedenovo Biotechnology Co., Ltd. (Guangzhou, China) following the vendor’s recommended protocol. The sequencing data have been deposited in the NCBI Sequence Read Archive and are accessible through NCBI BioProject Acc. No. PRJNA606858.

### Alignment and Identification of Small RNAs

Clean reads were filtered from raw reads by removal of adapters, ambiguous reads and low-quality reads (Berezikov et al., 2006). All of these reads were aligned with the small RNAs from the GenBank database Release_236.0 (Benson et al., 2008) and Rfam database Release_14.1 (Griffiths-Jones et al., 2005) to identify and remove ribosomal RNA (rRNA), small nuclear RNA (scRNA), small nucleolar RNA (snoRNA), small nuclear RNA (snRNA), and transfer RNA (tRNA) using BLAST+ (v2.10.0). Meanwhile, each detected read was also mapped to the *R. idaeus* assembled transcriptome sequenced by our laboratory (NCBI BioProject Acc. No. PRJNA606819), as the RNAs mapped to expressed sequence tags might be fragments from miRNA degradation. For the identification of known miRNAs, clean reads were searched against miRBase Release_22.1 by alignment with other species (Griffiths-Jones et al., 2006). Known miRNAs are counted at the family level, and miRNAs with less than 2 mismatches are categorized as the same family (Liang et al., 2010). miRNA^∗^ is here defined as the strand with lower frequency in two complementary strands. Reads that did not obtain a match in the above databases were defined as unclassified reads and were discarded.

### Prediction of Novel miRNAs

Unannotated reads obtained from clean reads after removal of rRNA, scRNA, snoRNA, snRNA, tRNA, and known miRNA mapped to the *R. idaeus* assembled transcriptome were used to identify novel miRNAs according to their hairpin structures predicted by software Mireap_V0.2. Novel miRNA predictions were conducted based on the following principles: (1) minimal and maximal miRNA sequence length of 18 and 35 nucleotides, respectively; (2) minimal and maximal miRNA reference sequence length of 20 and 23 nucleotides, respectively; (3) minimal and maximal space between miRNA and miRNA^∗^ of 16 and 300 nucleotides, respectively; (4) copy number of miRNAs on the reference of less than 20; (5) free energy allowed for a miRNA precursor not exceeding −18 kcal/mol; (6) maximal asymmetry of miRNA/miRNA^∗^ duplex of four nucleotides, with the bulge between the miRNA and miRNA^∗^ also being no more than four nucleotides; and (7) flank sequence length of miRNA precursor of 20 nucleotides (Meyers et al., 2008; Min and Yoon, 2010). All of these predicted miRNAs were summarized, and duplicates were deleted from the three different organs of the plant samples.

### Differential Expression of MiRNAs Between Three Libraries

The miRNA expression level was calculated and normalized to transcripts per million (TPM) (Buermans et al., 2010). The formula for TPM is as follows:

$$\text{TPM}=\text{ActualmiRNAcounts}/\text{Totalcountsofcleanreads}\times 10^{6}.$$

To identify differentially expressed transcripts across samples or groups, the edgeR package^2^ was used (Robinson et al., 2010). Based on the average TPM, as calculated from the three replicates of each organ, we considered mRNA with a fold change ≥ 2 and a false discovery rate (FDR) < 0.05 in a comparison as significantly differentially expressed genes (DEGs), while a fold change ≥ 2 and *P* < 0.05 was used for the miRNA.

### Prediction of Target Genes

Based on the sequences of known and novel miRNAs, the putative target genes were predicted by the software PatMatch v_1.2 (Yan et al., 2005) with the following parameters: (1) no more than two adjacent mismatches in the miRNA/target duplex, (2) no more than four mismatches between the sRNA and target (G-U bases count as 0.5 mismatches), (3) no adjacent mismatches in positions 2–12 (from 5′ of miRNA) of the miRNA/target duplex, (4) no mismatches in positions 10–11 of the miRNA/target duplex, (5) no more than 2.5 mismatches in positions 2–12 of the miRNA/target duplex, and (6) minimum free energy (MFE) of the miRNA/target duplex should be greater than or equal to 74% of its miRNA bound to a perfect complement.

### Analysis of GO and Pathway Enrichment

To analyze the molecular function, cellular components, and biological processes, we utilized GO enrichment to provide GO terms for both common miRNAs and DEGs compared with the background transcriptome (Consortium, 2004). Target genes were mapped to GO terms in the GO database^3^. These terms were summarized and classified by their function and location. Then, GO terms that were significantly enriched compared with the background genome were verified by a hypergeometric test, and *P*-values were calculated by the following formula (Rhee et al., 2008):

$$\text{P}=1-\sum_{i=0}^{m-1}\frac{\left(\begin{array}{c} M\\ i \end{array}\right)\,\left(\begin{array}{c} N-M\\ n-i \end{array}\right)}{\left(\begin{array}{c} N\\ n \end{array}\right)}$$

where *N* represents the number of all genes with GO annotations, *M* is the total number of annotated genes to the specific GO terms, and m is the number of DEGs in *M*. The *P*-value was FDR-corrected, and an FDR of ≤0.05 was used as the threshold.

Different genes playing various roles regulate biological functions. Further relative gene functions, such as metabolic pathways or signal transduction pathways, were identified by mapping to the Kyoto Encyclopedia of Genes and Genomes (KEGG) database: a major public pathway-related database. The method for calculating the *P*-value was the same as that in used in the GO analysis (Kanehisa et al., 2007).

### Stem-Loop qRT-PCR Identification

Stem-loop primers were designed for the qRT-PCR as described (Chen et al., 2005). The primers are shown in Supplementary Table S1. To verify the relative expression of the miRNAs in the root, stem, and leaf tissues, the reverse transcriptase reaction was performed using a HiFiScript cDNA Synthesis Kit (CW2569M, CWBIO, Beijing, China). The 20 μL mixture contained 0.5 μM each of dNTPs, stem-loop primers, 5 μg RNA template, 10 mM DTT, 200 U HiFiScript, and RNase-free water. Then, reverse transcription products were mixed with TB Green Premix Ex Taq (Tli RNaseH Plus, TaKaRa, Dalian, China) in a 96-well plate to start the real-time PCR reaction by a Roche LightCycler 480 II system using the following conditions: an initial denaturation step for 30 s at 95°C, 40 cycles of denaturation for 5 s at 95°C, 30 s at 60°C for annealing, and a 30 s extension at 72°C. The relative expression of miRNAs was calculated based on the abundance of the reference gene U6 snRNA. Then, the 2^–Δ^ ^ΔCT^ method was adopted to assess the relative miRNA expression from the qRT-PCR experiments (Livak and Schmittgen, 2001).

## Results

### High-Throughput Sequencing of Small RNAs From Raspberry

**FIGURE 1:**
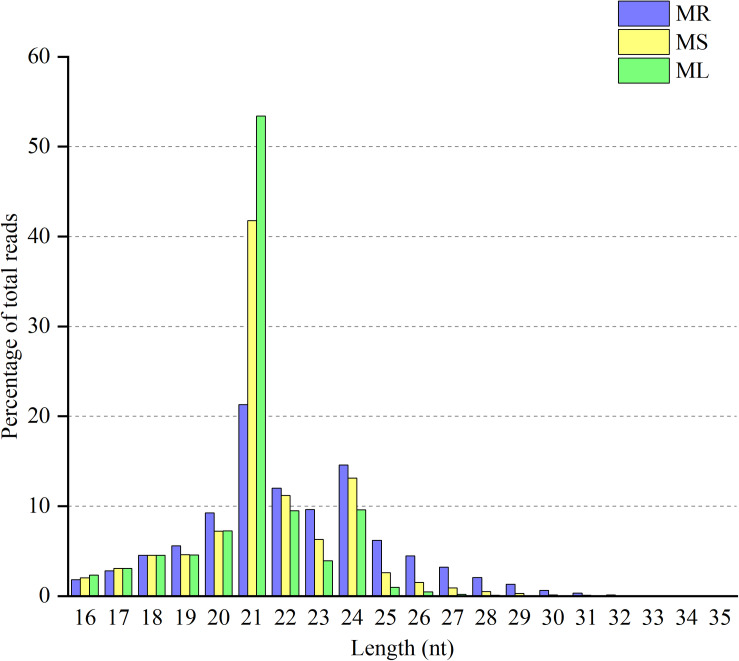
Distribution of clean reads with different sequence lengths according to their total reads in the root (MR), stem (MS), and leaf (ML) tissues.

The goal of this study was to identify miRNAs and predict their targets in three different organs in raspberry. Therefore, we performed high-throughput deep sequencing through the Illumina sequencing platform to construct small RNA libraries from the roots, stems, and leaves. More than 41,000,000 raw reads of total three replicates for each organ were identified from the constructed small RNA libraries, while the clean reads comprised over 80.88% of all reads in the appropriate sizes of 18−35 nt nucleotides without low quality or substandard reads (Table 1). In the small RNA libraries, the size distribution of the sequencing reads ranged between 18 to 35 nt and the 21 and 24 nt reads were the most enriched among all sequences from the three tissues. Interestingly, the 21 nt reads were less abundant in the root than those in the stem and leaf, while the 24 nt reads were more frequently in the root (Figure 1). Furthermore, the third-most frequent read length was 22 nt (12.0%) in the root, followed by 23 nt (9.6%) in the roots, while 20 nt was more abundant in the stems (7.2%) and leaves (7.3%). Some of the reads sequencing for low reads (<2 times) in less than two replicates will be excluded as sequencing errors, while a few sequencing reads sequenced over one hundred times were estimated to be relatively highly expressed based on abundance.

**TABLE 1:** Statistics of small RNA libraries analyzed by high-throughput sequencing.

**Read Data**	**Count of Roots**	**Count of Stems**	**Count of Leaves**
Raw reads	43,592,284 (100%)	41,195,510 (100%)	42,359,090 (100%)
High quality reads	42,679,590 (97.90%)	40,552,426 (98.43%)	41,719,365 (98.48%)
3′-Adapter null	334,717 (0.77%)	332,588 (0.81%)	252,908 (0.60%)
Insert null	2,909,737 (6.67%)	965,480 (2.34%)	1,194,815 (2.82%)
5′-Adapter contaminants	987,061 (2.26%)	124,536 (0.30%)	237,403 (0.56%)
Smarter than 18 nt	3,187,628 (7.31%)	3,056,952 (7.42%)	3,751,109 (8.86%)
Poly A	1,527 (0.87%)	434 (0.41%)	528 (0.36%)
Clean reads	35,256,638 (80.88%)	36,068,491 (87.55%)	36,281,612 (85.65%)
Number of reads corresponding to miRNAs	3,227,690 (9.15%)	11,405,914 (31.62%)	15,798,529 (43.54%)
			

### Identification of Known miRNAs in Raspberry

**FIGURE 2:**
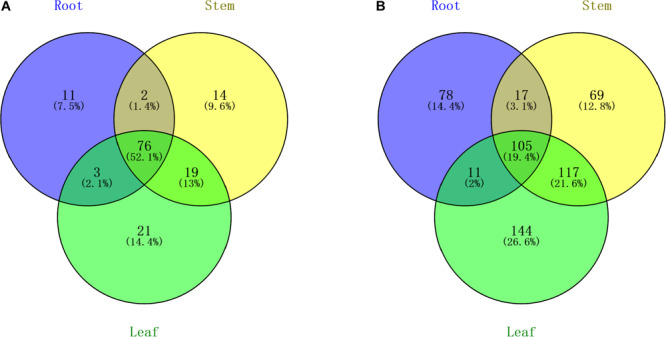
Venn diagram indicating the common and unique microRNAs in the root, stem, and leaf small RNA libraries. **(A)** Known miRNA distribution and **(B)** novel miRNA distribution.

All of the small RNA reads containing low-quality, 5′ adapter contaminants, 3′ adapter null, insert nulls, and polyA regions were removed to obtain clean reads of 18−35 nucleotides. Then, more than 35,000,000 clean reads of three replicates for each tissue were mapped to GenBank and Rfam to annotate the categories of the non-coding RNAs, including rRNA, snRNA, snoRNA, and tRNA. The rRNAs were abundant in the mapping results among the roots (11.66%), stems (12.15%), and leaves (8.14%) (Supplementary Table S2). The numbers of all annotated known and novel miRNAs distributed among the three organs of raspberry are shown in Figure 2. Next, all of these annotated RNAs were removed from the clean reads for the identification of known miRNAs through conservative sequence alignment with other species in miRbase.

Through mapping to the miRbase database, we identified 92 known miRNAs extracted from a total of 3,227,690 reads in the root, and the number of known miRNAs detected in the stem and leaf were 111 and 119, respectively. The names of known miRNAs followed the rules that “x” shows that miRNA is processed from the 5′ arm of the precursor, while “y” indicates the 3′ arm of the precursor. The detailed information of miRNA are shown in Supplementary Table S3. The length distribution of known miRNAs is from 18 to 35, while the most miRNAs with high expression (>100 in single organ) are 21 nt in length. The hairpin structure of 49 miRNAs was successfully predicted using mireap_v0.2, the length of miRNA hairpin is from 79 to 349 nt. According to previous reports, the MFEI of a given miRNA precursor tends to be higher than the values of the tRNAs, rRNAs, mRNAs, and even random sequences (Zhang et al., 2006b). Therefore, comparing the differences in MFEI of both becomes an effective way to distinguish precursors from miRNAs or other types of RNAs. The minimal folding free energy index (MFEI) of hairpin structures ranges from 0.5 to 1.41, and over 90% of the MFEIs of miRNAs are greater than 0.85. Moreover, we identified a total of 24 miRNA-miRNA^∗^ pairs based on three replicates of each organ, which made up about 16% of all miRNAs; these miRNA^∗^ are also shown in Supplementary Table S3 and were counted in the total number of known miRNAs. To analyze these miRNA counts, we found that there were more miRNAs from the 5′ arm of the hairpin structure than from the 3′ arm. Moreover, most of the miRNAs^∗^ were expressed at relatively low levels (<100), while some miRNAs^∗^, such as miR408^∗^ and miR398^∗^, had a higher level among all tissues.

**FIGURE 3:**
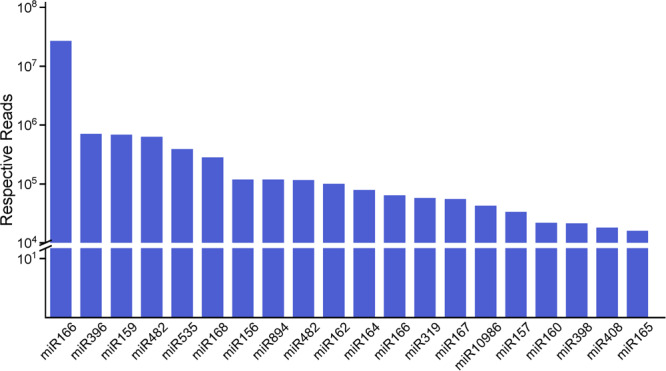
The top 20 miRNA families and their respective reads among three organs. The miR166 family has the highest number of respective reads among all of the miRNA families.

We analyzed the number of reads for known miRNAs and found that the expression frequency of these miRNAs was highly variable. In this research, we count miRNAs at the family level. Shifted sequences at that locus and shortened ones (total mismatches < 2) are also included in miRNA family frequency. Some of the miRNAs had a higher expression level in the stems, e.g., miR157 and miR319, while some other miRNAs were enriched in the roots or leaves compared with the stems (Supplementary Table S4). Some miRNAs were only detected in a particular tissue, showing that certain miRNAs are tissue specific. We describe the top 20 largest miRNA family reads among all of the miRNAs shown in Figure 3. These miRNA families are characterized by the presence of numerous miRNAs, as expected. Most of the highly expressed miRNAs, such as miR166, miR159, miR396 and some other miRNA families, are highly conserved in diverse plant species, suggesting that these representative miRNAs regulate similar pathways in widespread species. Also, miR166 have 52 family members, which is just below that of miR156 with 56 family members. The number of family members in miR159 and miR396 are, respectively, 41 and 51. Based on the frequency analysis, some pairs of miRNAs could actually be determined as being miRNA or miRNA^∗^, such as miR168, miR396, and miR319. Several miRNAs and miRNAs^∗^ were both relatively highly expressed, such as miR166 and miR408, and thus, are likely to both be functional. Nevertheless, there were still 69 known miRNAs present in different tissues at low abundances (<100); most of these miRNAs have unknown functions in raspberry.

### Prediction of Novel miRNAs in Raspberry

The small RNA libraries with 18 to 35 nucleotides from the three organs were searched for potential novel miRNAs. Reads were mapped to the assembled transcriptome of raspberry to discover correct novel miRNA precursors, and the secondary structures of these novel miRNAs for stable stem-loop hairpins were also predicted. The lengths of the miRNA precursors varied from 52 to 368 nucleotides, with an average of 160.4. Using Mireap_V0.2 software, a total of 542 types of novel miRNAs were identified, and a list of novel miRNA sequences, hairpin MFEIs, precursor sequences, and structures are shown in Supplementary Table S5. Among these 542 novel miRNA precursors, the MEFI values ranged from 0.28 to 1.76, and the average value was 0.75.

**FIGURE 4:**
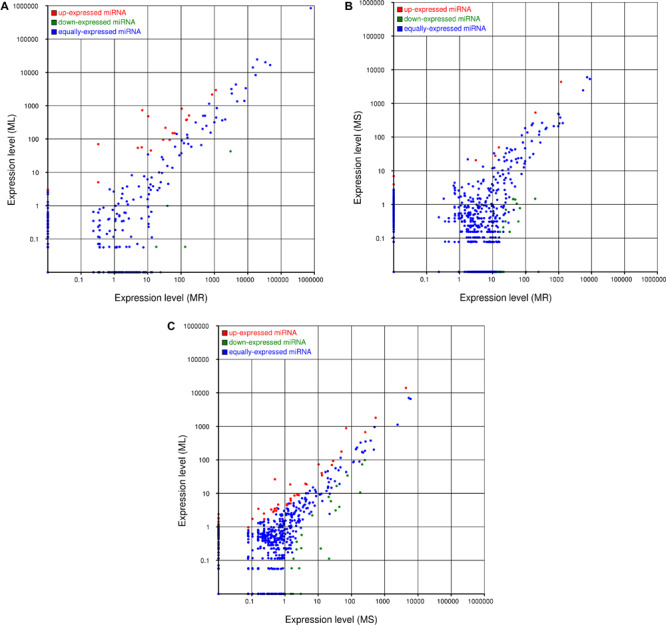
Comparisons of the different expression patterns of novel miRNAs between each pair of organs. Each point in the scatter plots represents one miRNA. The *x*-axis and *y*-axis individually show the expression levels between two libraries. Red dots indicate more abundant expression in the *y*-axis library (fold change > 1, *P* < 0.05); blue dots indicate equal expression between two libraries (–1 < fold change < 1, *P* > 0.05), and green dots indicate less enrichment in the *y*-axis library (fold change < –1, *P* < 0.05). **(A)** Expression in the root (MR) vs. expression in the leaf (ML), **(B)** expression in the root (MR) vs. expression in the stem (MS), and **(C)** expression in the stem (MS) vs. expression in the leaf (ML).

The expression levels of the novel miRNAs among the MR, MS, and ML libraries were compared (Figure 4). Based on Figure 4, we can conclude that most of the novel miRNAs were equally expressed among the three libraries, while several of the differentially expressed novel miRNAs deserve further attention to discern their functions. The distribution of the expression levels of the novel miRNAs was roughly similar to the annotated miRNAs. Some novel miRNAs, for example, novel_m0210, novel_m0825, novel_m0138, and novel_m0813, were barely expressed in the roots, but were highly expressed in the stems and leaves (Supplementary Table S6). Some novel miRNAs could be confirmed as novel miRNAs^∗^, such as novel_m1339, novel_m1058, and novel_m1130. However, it was difficult to detect novel miRNAs with multiple copies (count > 200) that were significantly more highly expressed in the roots than in the stems or leaves. These differentially expressed novel miRNAs may play important and specific potential regulatory roles in the different organs of raspberry. Also, compared with the known miRNAs, most of the novel miRNAs were expressed at lower levels. Some of the novel miRNA levels were comparable with the conserved miRNAs that were expressed in the different tissues; for example, novel-m0665-3p had 8862, 5298, and 7035 TPM on average in the root, stem, and leaf tissues, respectively. Additionally, a few novel miRNAs showed variation in expression among the tissues. We confirmed that novel-m0138 had a higher level of expression in the leaves in comparison with the roots and stems, while novel-m0310 was much more abundant in the roots. This high level of expression of novel miRNAs implies that they may have critical roles in raspberry development or other physiological processes.

### Differential Expression of miRNAs Among Three Organs in Raspberry

To identify the differentially expressed miRNAs in raspberry, the normalized expressions of the miRNAs in each organ were compared. The edgeR package was used to identify significantly differentially expressed miRNAs with a fold change ≥ 2 and *P* < 0.05. In the three comparisons, there were 59, 112, and 99 differentially expressed miRNAs in the roots vs. stems, stems vs. leaves, and roots vs. leaves, respectively (Supplementary Tables S7–S9). Among them, we found that 39, 79, and 74 differentially expressed miRNAs were novel miRNAs. Compared with the root, 22 and 57 miRNAs were up-regulated in the stems and leaves, and 37 and 42 miRNAs were down-regulated, respectively. However, in the stems vs. leaves, 70 miRNAs were up-regulated, and 42 miRNAs were down-regulated. These results suggested that both known and novel miRNAs may play specific but important roles in particular raspberry tissues.

### Potential Targets of Known and Novel miRNAs in Raspberry

The known and novel miRNAs were all found to have corresponding putative target genes. We used PatMatch (V1.2) to predict potential miRNA targets and their primary functions following the appropriate rules and steps. In total, 12,394 target sites of 8,907 target genes were predicted for known miRNAs, while 7,127 target sites of 4,823 target genes were obtained for novel miRNAs (Supplementary Tables S10, S11). A large proportion of these target genes have specific or presumed functions, and these target genes are involved in the regulation of diverse metabolic processes. The target sites are located in the coding regions.

A total of 13,730 target genes and 19,521 target sites among the three organs were subjected to GO and pathway analyses, and targets enrichment results of both known miRNAs and novel miRNAs are shown in Supplementary Tables S10, S11. For the GO analysis, the target genes were found to be related to nucleoside-triphosphatase activity (GO:0017111), protein-lysine N-methyltransferase activity (GO:0016279), and Ras GTPase binding (GO:0017016) in the roots, while the target genes were enriched in functions of cofactor binding (GO:0048037), motor activity (GO:0003774), and oxidoreductase activity, acting on the CH − CH group of donors, and with NAD or NADP as acceptor (GO:0016628) in both the stems and leaves. Some miRNA families such as miR157, miR395, and miR319 had more than two target sites, suggesting that these miRNAs are functionally divergent. Similarly, the same gene was also targeted by several miRNAs. These targets for the conserved and novel miRNAs have diverse functions, and their regulatory roles in raspberry need to be further studied.

**FIGURE 5:**
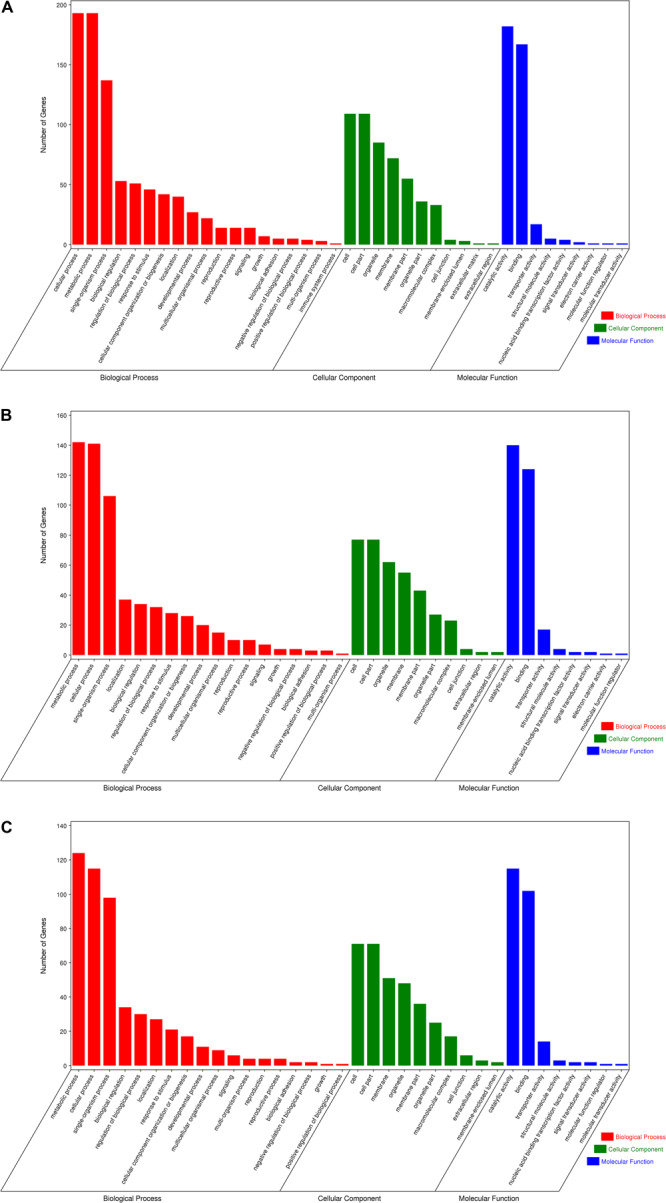
Gene ontology of the predicted targets for differentially expressed miRNAs. Classification of miRNA target genes was performed according to biological processes, cellular components, and molecular functions. **(A)** GO terms for root vs. leaf, **(B)** GO terms for root vs. stem, **(C)** GO terms for stem vs. leaf.

Gene Ontology enrichment analysis can help us grasp the distribution of the targets of differentially expressed miRNA in terms of biological processes, cellular components, and molecular functions. The distribution results between each pair of groups are shown in Figure 5. The differentially expressed miRNA targets between the roots and stems were abundant in cellular processes, metabolic processes, and single-organism processes within biological processes; this might be due to the regulatory role of miRNAs in plants (Zhang et al., 2006a). The targets of these miRNAs are mainly located in the cells, membranes, and organelles, and function in binding, catalysis, and transporter activity. Following GO analysis, we used KEGG to construct a pathway enrichment of the predicted miRNA target genes. Many metabolic networks were found to be involved, including plant–pathogen interaction, lipid metabolism, amino acid metabolism, carbohydrate metabolism, energy metabolism, nitrogen metabolism, and signal transduction.

### Quantitative PCR Expression Analysis

Quantitative real-time (qRT)-PCR analysis was used to confirm the relative expression of some significant differential miRNAs in the small RNA libraries. The potential relationships between miRNA expression and their functions in the organs were also explored. Total RNA was extracted from the stems, roots, and leaves as the template for reverse transcription. The sequences of the mature miRNA were confirmed through general PCR, and the abundance of mature miRNA was calculated by stem-loop qRT-PCR analysis. The technique mentioned above was described in the Materials and Methods section.

**FIGURE 6:**
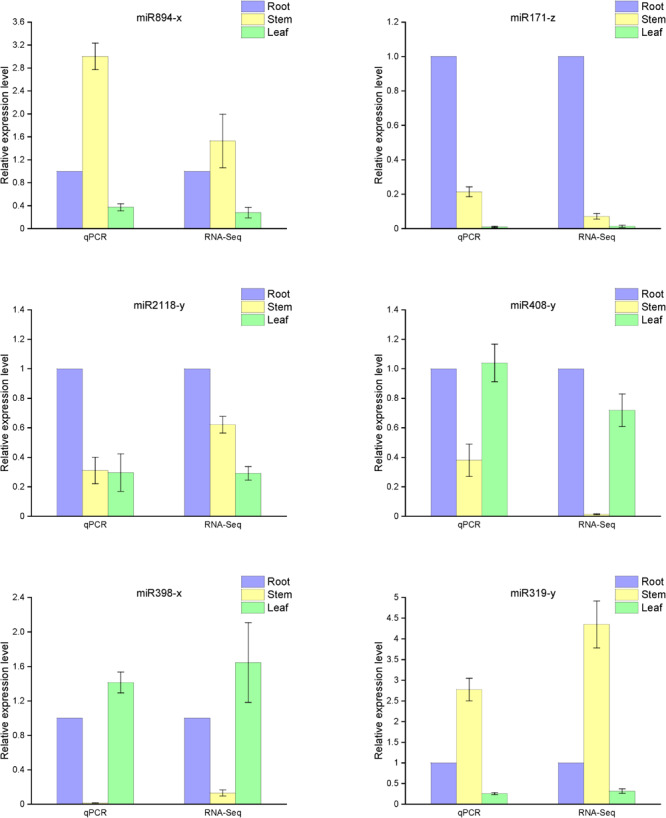
Expression levels of known miRNAs in the different organs of raspberry compared with the RNA-Seq results. The abundance of the random miRNAs was examined using stem-loop qRT-PCR. The expression levels of miRNAs were normalized to the level of U6 snRNA. Fold changes in the expression levels of miRNAs were estimated by the 2^–ΔΔCT^ method relative to the levels in the roots. Data are reported as mean ± standard error (SE) for three independent experiments.

Based on our analysis of the differences in miRNA function and mechanism among the three organs, we selected six representative microRNAs (miR894-x, miR171-z, miR2118-y, miR408-y, miR398-x, and miR319-y) to examine their relative expression in the various organs. As anticipated, the qRT-PCR results were consistent with the high-throughput sequencing data (Figure 6). Compared with the expression in the roots, miR408-y was relatively more highly expressed in both the stems and leaves, whereas miR2118-y and miR171-z exhibited lower enrichment in both the stems and leaves than in the roots. miR398-x was highly expressed in the leaves but was barely detectable in the stem, whereas miR319-y and miR894-x were barely present in the leaves but were more prominently expressed in the stems.

## Discussion

Since the discovery of miRNA at the beginning of the 21st century, the role of miRNA in modulating life activity functions has become a topic on increasing interest. miRNAs have a significant influence in regulating the growth and development of plant organs, and an increasing number of studies have demonstrated that specific miRNAs regulate almost all of the tissue and organ activities (Saliminejad et al., 2019). Developmental processes and responses to environmental changes may rely on fast and fine adjustments of mRNA or protein profiles, which can be partially achieved through miRNA-mediated control of mRNA decay or translation (Duarte et al., 2013). Thus, studying the functional mechanisms of different miRNAs in various plant tissues can help us to better understand the plant development process. Early seedling development is critical to successful stand establishment in plants, of which stage is under the control of miRNAs and their target genes. To date, researchers have detected miRNAs in an increasing number of species, but there is no relevant report of miRNAs in raspberry. In order to obtain common miRNAs and select miRNAs involved in the growth of raspberry seedlings, we provide a coherent approach for emphatically examining hundreds of miRNAs and their targets in root, stem and leaf tissues in the early development of raspberries. The identification of miRNAs was carried using Illumina HiSeq^TM^ 2500, a high-throughput sequencing platform, and the sequences and relative expression of these miRNAs were confirmed based on Chen et al. (2005) regarding the design of the stem-loop primers. In total, our study identified 147 known miRNAs and 542 novel miRNAs. We predicted their sequences, targets, and secondary structures among three different plant organs to gain a preliminary understanding of their states. These miRNAs were distributed in each of the examined organs of raspberry and should be studied further to confirm their functions and interfering mechanisms during growth and development.

Compared with the annotated miRNAs of miRBase, we found that the raspberry miRNAs were relatively conserved with those of higher model and closely relative plants, such as *Arabidopsis* (Wang et al., 2004), *Fragaria vesca* (Han et al., 2019), *Zea mays* (Aydinoglu and Lucas, 2019), and sweet cherry (Wang et al., 2019). A large number miRNAs, such as miR159, miR157 and miR394 have similar base composition with miRNAs in closely relative plants. From the miRNA family statistics, approximately 79 highly conserved miRNA families (total reads > 100) were detected; miR166 had the highest count of 26,783,298 reads, followed by miR396, miR159, miR482, and miR535. All of these miRNAs had hundreds of thousands of miRNA reads. Conversely, 183 families, including miR814, miR947, miR773, and miR444, had only one copy. Surprisingly, we found that the highly expressed miRNAs in raspberry showed similar expression patterns to the miRNAs in other plants, including *Arabidopsis*, tobacco, and soybeans; this may reflect the conserved functions of such miRNAs in different species. Additionally, some novel miRNAs were further investigated due to their high expression levels, for example, novel-m209, novel-m0665, novel-m0515, and novel-m0280. These miRNAs might have raspberry-specific roles in metabolic pathways or development processes.

It has been well documented that miRNAs play different but essential roles in different organs. miR156 affects the temporal expression changes of numerous genes during leaf development in rice and also participates in the root development of *A. thaliana* through regulating squamosa promotor binding protein-like (SPL) transcription factors (Xie et al., 2012; Yu et al., 2015). Our results indicate that miR156 has a high relative expression level in the root and leaf tissues, suggesting that this miRNA plays the same role in both organs of raspberry. To be certain of this, we explored target predictions of the miR156 family. Surprisingly, we found that one of the miR156 target genes was SPL-6. The miR394 family has been involved in salt and drought stress responses as a negative regulator, as it can also enhance the expression of argonaute-1, dsRNA-binding protein 4 (DRB4), and the RNA-binding protein gene dawdle (Song et al., 2013; Tian et al., 2018). In this study, we also found that DRB4 was modulated by the miR394 group, and we speculate that the same miRNA family will normally have the same target gene for comparison in raspberry and model plants.

The identification of target genes is a fundamental step for the determination of the biological function of miRNAs. On the basis of the perfect or near-perfect complementarity between a miRNA and its target mRNA, we can use BLAST analysis of miRNA mature sequences versus genomic sequences to identify the target genes (Colaiacovo et al., 2010). A large number of predicted targets have been confirmed by experimental approaches, such as Northern hybridization, 5′-RACE (rapid amplification of cDNA ends), and degradome analysis. In this study, we identified the targets of the differentially expressed known and novel miRNAs. The target annotation corroborated other research that had shown that many of the predicted targets are associated with transcription factors. We identified the predicted targets of the differentially expressed miRNAs encoding transcription factors, such as SPL (miR157-x), AS1 (miR10986-x), AGO2 (miR11293-x), and NF-VA3 (miR169-x) (Table 2). In many cases, one target was not only regulated by a single miRNA; for example, target prediction revealed that SPL-6 is simultaneously targeted by miR12140, miR156, miR157, miR529, and miR3699. Some differentially expressed miRNAs have two or more targets, allowing them to modulate multiple molecular mechanisms in different parts of the organism; for example, miR858-x, which is differentially expressed in roots and leaves, is predicted to regulate many transcription factors, such as MYB114, MYB9, and MYB46. Other miRNAs are predicted to only regulate a single target. For example, miR2118 enrichment in stems may regulate the disease resistance protein RGA2.

**TABLE 2:** Part of differentially expressed miRNAs and their predicted targets.

**miRNA**	**miRNA sequence**	**Length**	**Target Gene Family**
miR157-x	UUGACAGAAGAUAGAGAGCAC	21	SPL APRR INVE
miR384-x	UUGGCAUUCUGUCCACCUCC	20	CSC1-like protein
miR858-x	UUCGUUGUCUGUUCGACCUGA	21	Serine/threonine-protein kinase SRPK Transcription repressor MYB6-like NYNRIN-like protein
miR2118-y	UUUCCCAUGCCACCCAUUUCUA	22	Disease resistance protein RGA2
miR319-y	UUGGACUGAAGGGAGCUCCCU	21	Protein REVEILLE 7 Dof zinc finger protein Transcription factor GAMYB
miR395-y	CUGAAGUGUUUGGGGGAACUC	21	Threonine-protein kinase Sulfate transporter 3-like
miR398-x	GGAGCGACCUGAGAUCACAU	20	Serine/threonine-protein kinase CTR1 4-coumarate: coenzyme A ligase
miR171-z	UUGAGCCGCGCCAAUAUCACU	21	Scarecrow-like protein
miR1873-x	CAUGGUAUCAGAGCUGCAGGU	21	CTP synthase Copia protein
miR391-x	UACGCAGGAGAGAUGGCGCCGC	22	Calcium-transporting ATPase 8
miR408-y	AUGCACUGCCUCUUCCCUGGC	21	laccase-12-like
miR4405-y	AACAACCGACUUAGAACU	18	G-type lectin S-receptor-like 60S ribosomal protein L15 Homeobox-leucine zipper protein ATHB-8
miR5072-x	UCCCCAGCAGAGUCGCCA	18	ketone/zingerone synthase 2 Glutamine synthetase cytosolic isozyme Transducin/WD40 repeat-like superfamily protein
miR530-x	UCUGCAUUUGCACCUGCACCU	21	threonine-protein kinase RCH1 Nucleobase-ascorbate transporter 1
miR6118-y	UUUCCGAGUCCAGCCAUUCC	20	Disease resistance protein

**FIGURE 7:**
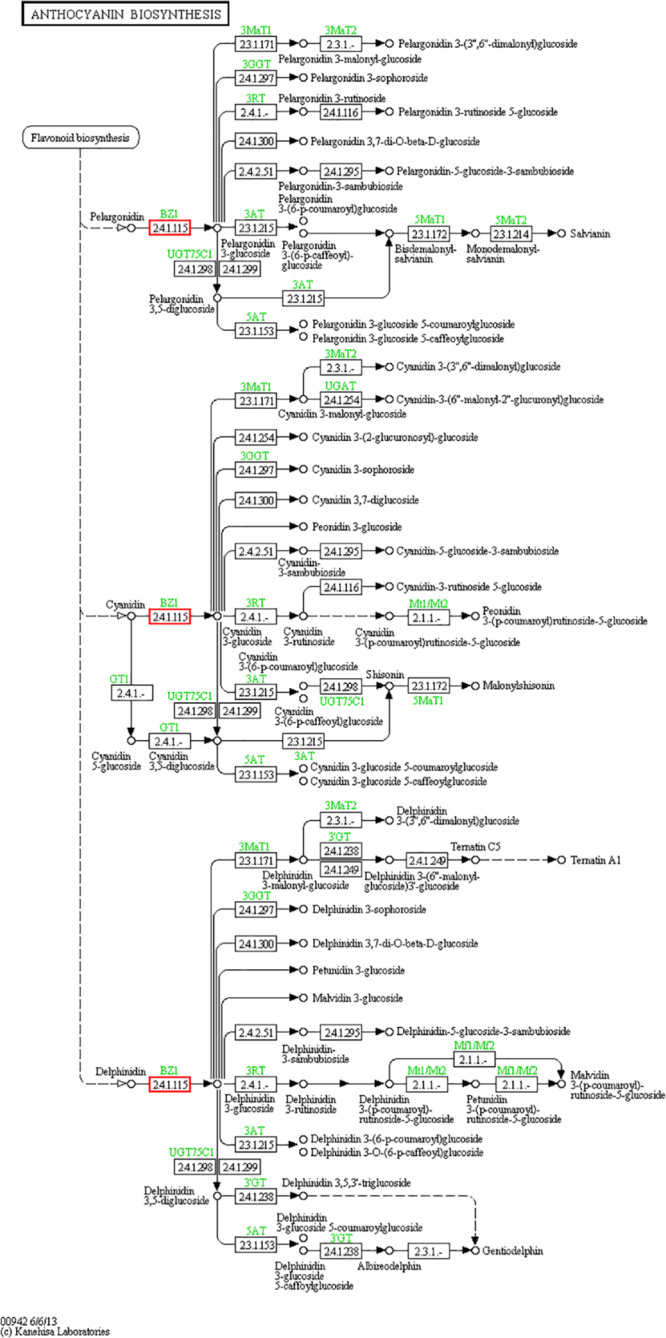
Anthocyanin biosynthesis pathway. The anthocyanin biosynthesis pathway was revealed by KEGG analysis. Small boxes denote proteins or enzymes (with EC numbers), while red boxes are the products encoded by candidate target genes. Specific genes or enzymes are indicated in green for some species. Small circles represent metabolites, and arrows represent the different metabolic pathways.

Particularly, the anthocyanins have been shown promising action against some diseases, such as diabetes (Gowd et al., 2017), cancer (Wang and Stoner, 2008) and Parkinson’s disease (Fan et al., 2018), both as treatments and dietary additives. Some raspberry extracts, such as salicylic acid, are also available as a raw material of some medicines or processed products, such as aspirin (Klessig, 2016) and contrast agents (Banerjee et al., 2018). For these reasons, it is important to better understand the miRNAs involved in regulating the biosynthesis and accumulation of such metabolites in raspberries. We searched for relevant target genes involved in the synthesis of anthocyanins and salicylic acid. First, we focused on anthocyanin biosynthesis (Figure 7, pathway ID: KO00942). We hypothesized that the miR858, miR5077, and miR5021 families are hypothesized to participate in the down-regulation of anthocyanin regulatory C1 protein from the target prediction, and miR2873 may negatively regulate anthocyanidin 3-*O*-glucosyltransferase expression. Among the miRNAs named above, the miR858 family has been reported to induce anthocyanin accumulation in tomatoes and activate anthocyanin biosynthetic pathways in *Arabidopsis* (Jia et al., 2015; Wang et al., 2016). Some miRNAs, such as miR4398, miR5207, miR5648, and miR774, have been inferred to be salicylic acid regulatory factors, affecting salicylic acid glucosyltransferase and its binding protein synthesis pathway. However, salicylic acid-related miRNAs have not yet been reported, providing an avenue of research for exploring miRNA-regulated salicylic acid synthesis. We also analyzed the database and concluded that some miRNAs such as miR1087, miR10195 and miR10200 are involved in resisting adverse environmental stress, and these miRNAs are related to metal ion sensitivity, cell wall permeability, and nutrient transport function. These miRNAs could probably assist target genes in the production of related proteins by changing their mode of expression to protect cells from salt-stress. Future research should focus on exploring functional miRNAs of raspberry in flower and fruit tissues or under salt-stress conditions.

In addition to the above, we focused on the miR171 family and their functions in the development of various organs of raspberry. miR171 has been reported as negatively regulating shoot branching, fruit formation, and the plant stress response through targeting scarecrow-like 6-II (SCL6) of the GRAS gene family (Wang et al., 2010; Lopez-Gomollon et al., 2012; Huang et al., 2017). In addition, some research has shown that miR171-z is involved in root development, as overexpressing miR171-z transgenic plants showed decreased primary root elongation and other pleiotropic phenotypes (Wang et al., 2010). For preliminarily inferring whether miR171-z plays the same role in raspberry, we analyzed the expression in raspberry. We discovered that other miR171 family numbers have roughly similar expression in the roots, stems, and leaves, however, miR171-z was much more abundant in the roots than in the stems and leaves, implying that miR171-z also plays a role in raspberry development. Additionally, miR171-z target gene prediction revealed that SCL-6 is one of the miR171-z targets, while miR171-x is forecasted to be involved in the synthesis of classical arabinogalactan protein. Thus, the miR171 family could have a regulatory role, participating in root-related metabolic synthesis.

In summary, we investigated both known and novel miRNAs from three organs in raspberry using the high-throughput sequencing technology. Stem-loop RT-PCR experiments were employed to confirm the expression of these miRNAs. Furthermore, the GO annotation and pathway analysis for predicted targets have implicated the putative roles of the abundant miRNAs among different organs in the same plant. However, one drawback of this study is that only three organs were sampled, and the flower and fruit samples were absent. Future research should focus on identifying miRNA in all organs during different development stages of floricane and primocane-fruiting raspberries. This will provide a panorama of miRNAs from different raspberry organs and development stages. It is also advantageous to elucidate the functional roles of miRNAs in raspberry. Notably, this study provides basic data for miRNA identification to promote further understanding of miRNA regulation in raspberry growth.

## Data Availability Statement

The datasets generated for this study can be found in the NCBI Bioproject: PRJNA606858 and PRJNA606819.

## Author Contributions

HY, LL, and JZ contributed to the design of the work. GY and HY analyzed the sequencing data and drafted the work and revised it critically for important intellectual content. GY, XG, and MJ detected the expression of miRNAs. HY approved the final version of this publication, and was accountable for all aspects of the work, ensuring that questions related to the accuracy or integrity of any part of the work were appropriately investigated and resolved.

## Conflict of Interest

The authors declare that the research was conducted in the absence of any commercial or financial relationships that could be construed as a potential conflict of interest.

## Footnotes

1 http://www.mirbase.org/index.shtml

2 http://www.bioconductor.org/packages/release/bioc/html/edgeR.html

3 http://geneontology.org
